# P-LAP/IRAP-induced cell proliferation and glucose uptake in endometrial carcinoma cells via insulin receptor signaling

**DOI:** 10.1186/1471-2407-7-15

**Published:** 2007-01-19

**Authors:** Kiyosumi Shibata, Hiroaki Kajiyama, Kazuhiko Ino, Akihiro Nawa, Seiji Nomura, Shigehiko Mizutani, Fumitaka Kikkawa

**Affiliations:** 1 Department of Obstetrics and Gynecology, Nagoya University Graduate School of Medicine, Nagoya, Japan

## Abstract

**Background:**

Hyperglycemia or hyperinsulinemia contributes to poorer endometrial cancer survival. It was shown that P-LAP/IRAP translocates to the plasma membrane in response to insulin stimulation. Recently, we demonstrated that P-LAP/IRAP is associated with a poor prognosis in endometrial adenocarcinoma patients. The aim of this study was to examine whether the malignant potential of endometrial cancer enhanced by P-LAP/IRAP is due to increased glucose uptake via the P-LAP/IRAP-mediated activation of insulin signaling.

**Methods:**

We transfected P-LAP/IRAP cDNA into A-MEC cells (endometrial adenocarcinoma cell line), and A-MEC-LAP cells expressed a remarkably high level of GLUT4 proteins.

**Results:**

^3^H-2-deoxyglucose uptake which responds to insulin in A-MEC-LAP cells was significantly higher than that of A-MEC-pc cells. A-MEC-LAP cells exhibited a significant growth-stimulatory effect compared to A-MEC-pc cells. A-MEC-LAP cells expressed a remarkably high level of p85PI3K protein compared to A-MEC-pc cells, and showed a higher degree of AKT phosphorylation by insulin stimulation.

**Conclusion:**

In summary, P-LAP/IRAP was involved in the increasing malignant potential of endometrial cancer mediated by insulin. P-LAP/IRAP was suggested to be a potential new target of molecular-targeted therapy for endometrial cancer.

## Background

Endometrial carcinoma is the most common gynecological malignancy in the United States. Recently, the incidence of this disease has been increasing in Japan. Several personal and lifestyle-related characteristics, such as age, obesity, diabetes, and estrogen therapy, have been identified as risk factors for developing endometrial cancer [1]. A recent study showed that diabetes is associated with reduced survival after endometrial cancer, independent of tumor stage and grade [2]. This finding suggested the possibility of a diabetes-related condition, such as hyperglycemia or hyperinsulinemia, contributing to reduced survival in endometrial cancer patients. Furthermore, an *in vitro *study showed that endometrial cancer cells have high-affinity binding sites for insulin and proliferate in response to insulin exposure [3]. Several studies have shown that insulin signal transduction proteins, in particular the insulin receptor (IR) and IRS-1, may also contribute to the development of various tumors [4,5].

Placental leucine aminopeptidase (P-LAP) is a cell surface aminopeptidase, and is a synonym for oxytocinase [6]. P-LAP is also referred to as insulin-regulated membrane aminopeptidase (IRAP), associated with the glucose transporter 4 (GLUT4) containing vesicle [7-9]. It was shown that P-LAP/IRAP translocates to the plasma membrane in response to insulin stimulation. Recently, we demonstrated that P-LAP/IRAP is present in both human endometrial adenocarcinoma tissues and cells, and that it acts as a regulator of carcinoma cell growth [10]. Furthermore, we demonstrated that P-LAP/IRAP is associated with a poor prognosis in human endometrial adenocarcinoma patients [11]. The aim of this study was to examine whether the proliferation of endometrial cancer enhanced by P-LAP/IRAP is due to increased glucose uptake via the P-LAP/IRAP-mediated activation of insulin signaling.

## Methods

### Tissues

Tissue specimens were obtained from patients who were surgically treated at Nagoya University Hospital. All tissue samples were fixed in 10% formalin, embedded in paraffin, and routinely stained with hematoxylin and eosin for histological examination. Endometrial adenocarcinomas were graded according to the criteria of the World Health Organization and classified as grade 1 (well-differentiated), grade 2 (moderately- differentiated), or grade 3 (poorly-differentiated). The relevant institutional review boards approved the study and informed consent from which all patients signed.

### Cell lines and culture conditions

We used 3 human endometrial endometrioid adenocarcinoma cell lines (A-MEC, HEC1A, and Ishikawa). A-MEC was a kind gift from Aichi Medical University, Ishikawa from Dr. M. Nishida (Kasumigaura Hospital, Ibaragi, Japan), and HEC1A from Professor H. Kuramoto (Kitazato University, Kanagawa, Japan). Cells were maintained in RPMI 1640 medium (Sigma Chemical Co., St. Louis, MO) supplemented with 10% fetal calf serum (FCS) and penicillin-streptomycin. These cells were incubated at 37 C in a humidified atmosphere of 5% CO_2_.

### Immunohistochemistry

Immunohistochemical staining was performed using the avidin-biotin immunoperoxidase technique (Histofine SAB-PO kit, Nichirei, Tokyo, Japan). Sections were cut at a thickness of 4 μm, and immunostained by the streptavidin/biotin/peroxidase method. Deparaffinized sections in 0.01 M citrate buffer were treated three times for 5 min each at 90 C and 750 W using an H2500 microwave oven. Sections were incubated in 0.3 % hydrogen peroxide for 20 min and then further incubated with 10 % normal goat serum for 10 min to block the endogenous peroxidase activity and non-specific immunoglobulin binding, respectively. Rabbit polyclonal antibody (sc-710, Santa Cruz Biotechnology, Inc., Santa Cruz, CA) at a 1:100 dilution was used for insulin receptor (IR) a, rabbit polyclonal antibody (Upstate, Inc., Lake Placid, NY) at 1:200 dilution was used for insulin receptor substrate-1 (IRS-1), rabbit polyclonal antibody (Santa Cruz Biotechnology, Inc., Santa Cruz, CA) at a 1:50 dilution was used for GLUT4, and rabbit polyclonal antibody at 1: 100 was used for P-LAP/IRAP. They were added to the tissue sections and incubated for 1 h in a moist chamber at room temperature. Binding of the antibodies was followed with biotinylated goat anti-rabbit IgG and horse-radish peroxidase-conjugated streptavidin (Histofine SAB-PO, Nichirei). Chromogenic development was performed by immersion of the sections in 3-amino-9-ethylcarbazole (AEC, Nichirei). The slides were counterstained with Mayer's hematoxylin.

### Plasmid construction and transfection

The eukariotic expression vector pcDNA3.1(-) (Invitrogen Japan K.K., Tokyo, Japan) was used to drive the expression of inserted P-LAP/IRAP cDNA. Transfections were carried out using Lipofectamine according to the manufacturer's instructions (Invitrogen Japan K.K., Tokyo, Japan). A-MEC cells were transfected with pcDNA3.1(-) (A-MEC-pc) or pcDNA3.1(-) inserted with P-LAP/IRAP cDNA (A-MEC-LAP). Stable transfectants were selected by growth in medium supplemented with 400 mg/ml of G418 (Sigma Chemical Co., St. Louis, MO). Several hundred clones resistant to G418 were obtained and polyclonal cells from these transfectants were used in the following experiments to eliminate any effects that could be attributed to clonal variation.

### Western blotting

Cell lysates were electrophoresed on 7.5% sodium dodecyl sulfate polyacrylamide gel under reducing conditions. After electrophoresis, the proteins were transferred electrophoretically to an Immobilon membrane (Millipore, Bedford, MA, USA). After blocking, the membrane was incubated for 1 h with rabbit polyclonal antibody against human P-LAP/IRAP, GLUT4, insulin receptor, IRS-1, p-Ser307-IRS1 (Upstate Biotechnology, Lake Placid, NY), p-Tyr1158-IR(Abcam, Cambrige, UK), and mouse monoclonal antibody for phospho-AKT (587F11, Cell Signaling Technology, Inc.) and p85 PI-3 kinase (sc-1637, Santa Cruz Biotechnology, Inc., Santa Cruz, CA) The membrane was washed with TBS-T for 15 min three times, and then incubated with peroxidase-conjugated goat anti-rabbit IgG for 1 h. After washing with TBS-T, the membrane was subjected to ECL-Western blotting detecting reagent (Amersham Biosciences K.K., Tokyo, Japan).

### Cellular glucose uptake in P-LAP/IRAP transfection cells

A-MEC-pc and A-MEC-LAP cells were cultured in 12-well plates. Twenty-four h after plating, the cells were serum-starved for 2 h, then incubated in either the presence or absence of 10 nM insulin for 20 min, followed by the addition of 2-deoxy-D-2,6-^3^H-glucose to a final concentration of 1 μCi/ml. Insulin was purchased from Sigma (Japan).

### Cell growth analysis

To evaluate the effect of insulin on cell proliferation, cells were seeded in triplicate in 96-well plates at a density of 5000 cells in a volume of 200 ml. Twenty-four h after plating, insulin was added to the culture medium at concentrations ranging from 10^-8 ^to 10^-7 ^M in the presence 5% FCS. Cell viability after 48 h was assayed using a modified tetrazolium salt 3-(4, 5-dimethylthiazol-2-yl)-2, 5-diphenyltetrazolium bromide assay with the Cell Titer 96 Aqueous One Solution Cell Proliferation Assay kit (Promega Corp., Tokyo, Japan) according to the manufacturer's instructions. Absorbance was measured at 490 nm by a microplate reader (Multiskan Bichromatic, Labsystems, Helsinki, Finland).

### Statistical analysis

Statistical comparisons among groups were performed using Student's *t*-test and ANOVA with Bonferroni corrections. Differences between groups were considered significant at *p *< 0.05.

## Results

### Expressions of P-LAP/IRAP, GLUT4, IR, and IRS-1 in endometrial carcinoma tissues and cell lines

**Figure 1:**
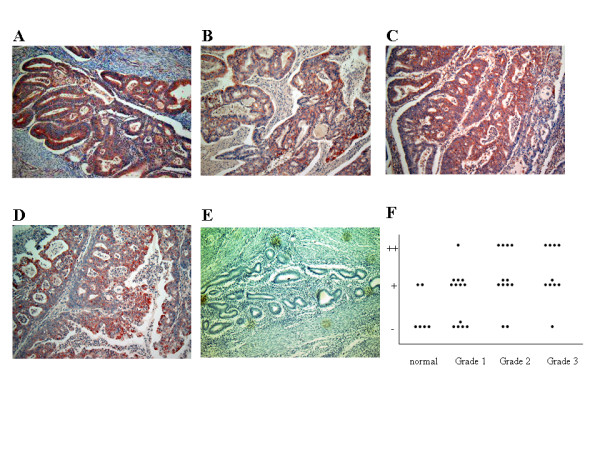
Immunochemical localizations of P-LAP/IRAP, GLUT4, IR, and IRS-1 in human endometrioid adenocarcinoma tissues (Magnification, × 200). A: P-LAP/IRAP in a grade 2 endometrioid adenocarcinoma. B: GLUT4. C: IR. D: IRS 1. Immunoreactivites of P-LAP/IRAP, GLUT4, IR, and IRS-1 were positive in carcinoma cells. E: Negative control (no primary antibody) in normal endometrial tissue.

**Figure 2:**
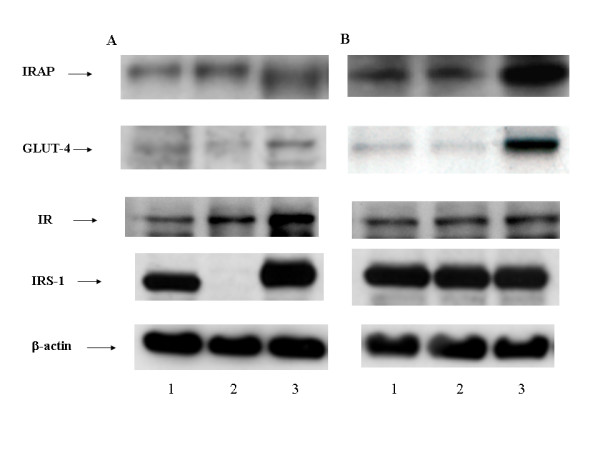
A. Expressions of P-LAP/IRAP, GLUT4, IR, and IRS-1 in three endometrial carcinoma cell lines. Lanes are as follows: lane 1, A-MEC cells; lane 2, HEC-1A cells; lane 3, Ishikawa cells. B. The effect of P-LAP/IRAP transfection on GLUT4, IR, and IRS-1 expressions in A-MEC cells. Lanes are as follows: lane 1, A-MEC cells; lane 2, A-MEC-pc cells; lane 3, A-MEC-LAP cells.

We first examined the expressions of P-LAP/IRAP, GLUT4, IR, and IRS-1 in human endometrial carcinoma tissues by immuohistochemistry. In 26 of 35 endometrial adenocarcinomas, tumor cells were P-LAP/IRAP-positive. In all positive tumor cells, P-LAP/IRAP staining was intense and localized at the periphery of the cytoplasm, close to the cell membrane (Fig. 1A). We showed a significant tendency of increased immunoreactivity with grade advancement in the previous report [10]. In 27 of 35 cases, tumor cells were GLUT4-positive. GLUT4 was stained in both the cell membrane and cytoplasm (Fig. 1B). The intensity of immunohistochemical staining was scored and plotted in Fig. 1F, showing the tendency of increased immunoreactivity with grade advancement. In 26 of 35 endometrial adenocarcinomas, tumor cells were IR-positive. IR was stained in the cell membrane (Fig. 1C). In 23 of 35 cases, tumor cells were IRS-1 positive. IRS-1 was stained in both the cell membrane and periphery of the cytoplasm (Fig. 1D). Neither the IR nor the IRS-1 staining levels showed any correlation with tumor grade advancement (data not shown). A-MEC, HEC-1A, and Ishikawa cells were all positive for P-LAP/IRAP and GLUT-4 on Western blotting. A-MEC cells showed the lowest expressions of P-LAP/IRAP and GLUT-4 among these cell lines. Ishikawa cells showed the highest expressions of P-LAP/IRAP and GLUT-4 among these cell lines (Fig. 2A). To investigate the effect of P-LAP/IRAP transfection on GLUT4, IR, and IRS-1 expressions in carcinoma cells, we overexpressed P-LAP/IRAP in A-MEC cells (A-MEC-LAP). While both parental A-MEC and expressed few GLUT4 proteins, A-MEC-LAP cells expressed a remarkably high level of GLUT4 proteins (Fig. 2B). Both IR and IRS-1 expressions in A-MEC-LAP cells were at similar levels compared with A-MEC and A-MEC-pc cells (Fig. 2B).

### Involvement of P-LAP/IRAP in glucose uptake in endometrial carcinoma cells

**Figure 3:**
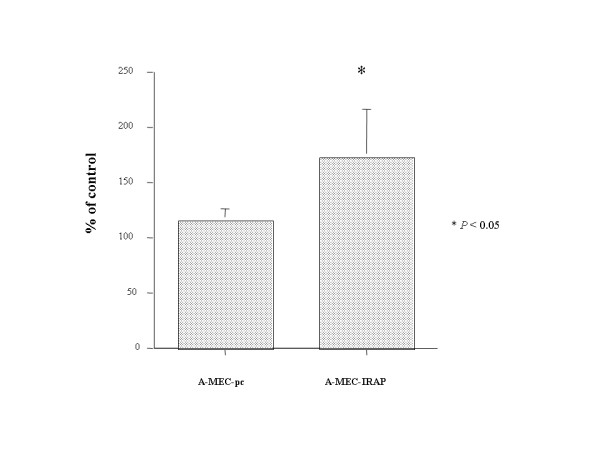
Cellular glucose uptake rates in A-MEC-pc and A-MEC-LAP cells. ^3^H-2-deoxyglucose uptake was measured as either basal or insulin-stimulated (10 nM insulin) for 30 min. Insulin-stimulated compared with basal rates in A-MEC-pc and A-MEC-LAP cells are shown. Data are expressed as the mean ± SEM of three separate experiments.

To determine whether the aberrant expression of P-LAP/IRAP influenced glucose uptake, insulin-stimulated rates of cellular glucose uptake were assessed in A-MEC-pc and A-MEC-LAP cells. As shown in Fig. 3, ^3^H-2-deoxyglucose uptake which responds to insulin in A-MEC-LAP cells was significantly higher than that of A-MEC-pc cells.

### Involvement of P-LAP/IRAP in insulin-induced cell growth

**Figure 4:**
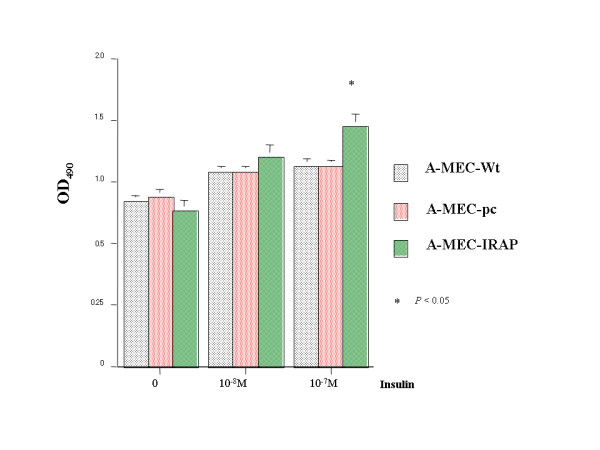
Effect of insulin on cell proliferation in A-MEC, A-MEC-pc, and A-MEC-LAP cells. Treatment with insulin increased cell proliferation at concentrations of 10^-8 ^and 10^-7 ^M. A-MEC-LAP cells exhibited a growth-stimulatory effect via insulin, and the addition of 10^-7 ^M of insulin led to a significant effect compared with A-MEC and A-MEC-pc cells (*p *< 0.05).

We next investigated the effects of insulin on cell proliferation in A-MEC, A-MEC-pc, and A-MEC-LAP cells. As shown in Fig. 4, the addition of insulin increased cell numbers in a dose-dependent manner in A-MEC cells. In particular, A-MEC-LAP cells exhibited a growth-stimulatory effect of insulin and the addition of 10^-7 ^M of insulin led to a significant effect compared to A-MEC and A-MEC-pc cells.

### The effect of P-LAP/IRAP transfection on insulin induced activation of the PI3K/AKT pathway in A-MEC cells

**Figure 5:**
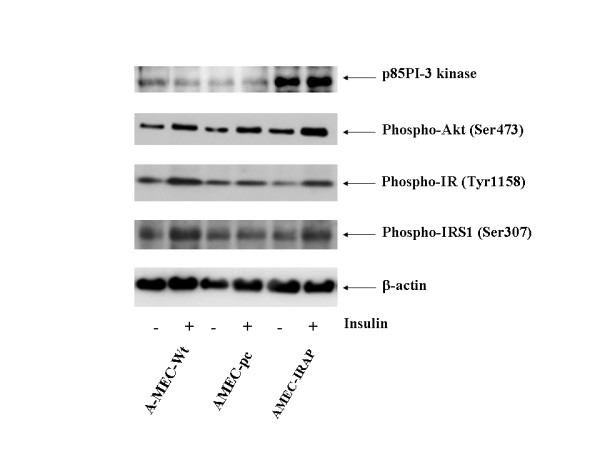
The effect of P-LAP/IRAP transfection on insulin induces activation of the PI3K/AKT pathway in A-MEC cells. The cells were stimulated on 10^-7 ^M insulin for 30 min in serum free medium. In A-MEC-LAP cells, with or without insulin, p85PI3K protein expression was increased compared with those of A-MEC and A-MEC-pc cells. In addition, AKT phosphorylation in the insulin-stimulated A-MEC-LAP cells was stronger compared with those in A-MEC and A-MEC-pc cells. Insulin stimulated IR and IRS1 phosphorylation were observed in all A-MEC, A-MEC-pc, and A-MEC-LAP cells to the same degree.

We next investigated whether the PI3K/AKT pathway was activated by insulin in A-MEC, A-MEC-pc, and A-MEC-LAP cells. A-MEC-LAP cells expressed a remarkably high level of p85PI3K protein compared with A-MEC or A-MEC-pc cells. Insulin stimulation enhanced AKT phosphorylation in all A-MEC, A-MEC-pc, and A-MEC-LAP cells, among which A-MEC-LAP cells showed the highest degree of AKT phosphorylation. Insulin stimulated IR and IRS1 phosphorylation were observed in all A-MEC, A-MEC-pc, and A-MEC-LAP cells to the same degree (Fig. 5).

### Blockade of the PI3K/Akt pathway inhibited the effect of insulin on A-MEC-LAP cell

**Figure 6:**
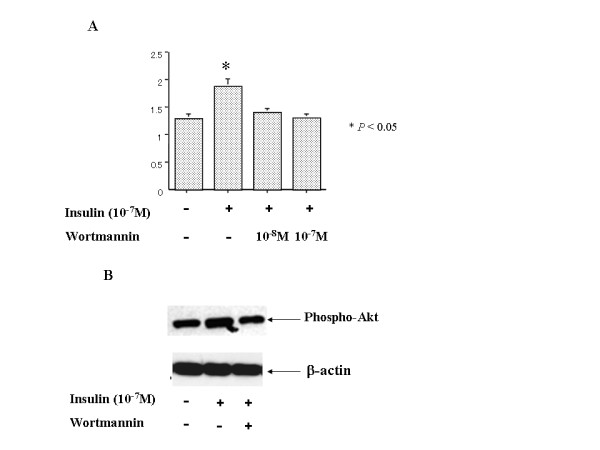
A: Effect of wortmannin pretreatment on the insulin-induced cell proliferation of A-MEC-LAP cells. The cells were pretreated with 10^-8 ^M or 10^-7 ^M wortmannin before 1 h stimulation with 10^-7 ^M insulin. Insulin-induced cell proliferation was significantly inhibited with 10^-8^M or 10^-7 ^M wortmannin. * *p *< 0.05 B: Effect of wortmannin pretreatment on insulin-induced AKT activation of A-MEC-LAP cells. Pretreatment with 10^-7 ^M wortmannin inhibited insulin-induced AKT activation in A-MEC-LAP cells.

The activation of AKT after treatment with insulin involves multiple pathways. To determine the functional role of PI3K/AKT activation in the insulin-induced cell proliferation of A-MEC-LAP cells, A-MEC-LAP cells were pre-treated with the PI3K inhibitor, wortmannin, 1 h before of insulin treatment. As shown in Fig. 6, the pre-treatment of A-MEC-LAP cells with wortmannin, significantly inhibited insulin-induced cell proliferation (Fig. 6A) and activation of AKT (Fig. 6B)(* *p *< 0.05).

## Discussion

Tumorigenesis is associated with enhanced cellular glucose uptake and increased metabolism. Indeed, both GLUT1 and GLUT4 are aberrantly expressed in many tumors; for example, overexpression of GLUT1 is found in breast, thyroid, gastric, and liver cancer [10-13]. GLUT4 is expressed in human astrocytic tumors, a subset of lung cancers, gastric cancer, and rhabdomyosarcoma [14-17]. Furthermore, a recent study showed that the tumor suppressor p53 down regulates GLUT1 and GLUT4 gene expression [18]. In previous studies, we demonstrated that P-LAP/IRAP was a poor prognostic factor of endometrial cancer, and that P-LAP/IRAP was involved in proliferation, anticancer agent resistance, and anti-apoptosis by a P-LAP/IRAP gene transfer experiment [11,12,19]. In this study, we showed the tendency of an increased GLUT4 immunoreactivity with the advancement of the tumor grade, and this tendency was similar to the results of P-LAP/IRAP. Diabetes and hyperglycemia are poor prognostic factors of endometrial cancer, and *in vitro *insulin responsiveness and enhanced proliferation of endometrial cancer cells have also been reported [1,2,13,14,20,21]. P-LAP/IRAP resides in GLUT4 vesicles, and is known to migrate to the cell membrane to facilitate glucose uptake into cells in response to insulin stimulation. In this study, we first demonstrated that P-LAP/IRAP gene transfer increased GLUT4 expression in endometrial cancer cell lines, with resulting increases in glucose uptake into cells and the cell proliferation capacity in response to insulin. Also, increased PI3K-AKT signal expression, a representative one in insulin signaling, was noted in the P-LAP/IRAP gene-transferred cells. The above results suggest the involvement of P-LAP/IRAP expression in insulin-induced carcinogenesis and a higher degree of malignancy of endometrial cancer cells. Recently, there have been several reports concerning activated AKT signal involvement in anticancer agent resistance [22-24]. We also reported the involvement of P-LAP/IRAP in anticancer agent resistance and anti-apoptosis. These results suggest that P-LAP/IRAP may also be involved in anticancer agent resistance via the activation of insulin signaling. We had postulated the involvement of enzyme activity in many cases of P-LAP/IRAP-induced increased proliferation and the anticancer agent resistance of endometrial cancer cells, and, in this study, we demonstrated the possibility that an increased glucose uptake into the cells by insulin also induces cell proliferation in endometrial cancer cells. Further studies on the mechanism of increased GLUT4 expression by P-LAP/IRAP gene transfer are needed in the future. In this regard, the involvement of hormonal treatment (e.g., estrogen and progesterone) and p53 in the regulation of GLUT4 expression has been reported [18,25], and their implications in P-LAP/IRAP expression must therefore be investigated.

In summary, P-LAP/IRAP was involved in the progression of endometrial cancer mediated by insulin signaling. P-LAP/IRAP was suggested to be a potential new target of molecular-targeted therapy for endometrial cancer.

## Conclusion

This work demonstrated that P-LAP/IRAP is involved in the increasing malignant potential of endometrial cancer mediated by insulin and to be a potential new target of molecular-targeted therapy for endometrial cancer.

## Competing interests

The author(s) declare that they have no competing interests.

## Authors' contributions

KS participated in study design, performed cell culture, transfection, and western blotting, drafted and revised the manuscript. HK investigated Cellular glucose uptake in P-LAP/IRAP transfection cells. KI participated in study design, performed immunohistochemical staining. AN participated in study design, performed western blotting. SN performed statistical analysis. SM participated in interpretation and critical review of the manuscript. FK participated in study design and coordination, supervision of experimental conduct and analysis, drafting and revision of the manuscript, and approved the final version. All authors have read and approved the final manuscript.

## Pre-publication history

The pre-publication history for this paper can be accessed here:
